# Screening for biomarkers of liver injury induced by *Polygonum multiflorum*: a targeted metabolomic study

**DOI:** 10.3389/fphar.2015.00217

**Published:** 2015-10-02

**Authors:** Qin Dong, Na Li, Qi Li, Cong-En Zhang, Wu-Wen Feng, Guang-Quan Li, Rui-Yu Li, Can Tu, Xue Han, Zhao-Fang Bai, Ya-Ming Zhang, Ming Niu, Zhi-Jie Ma, Xiao-He Xiao, Jia-Bo Wang

**Affiliations:** ^1^China Military Institute of Chinese Medicine, 302 Military HospitalBeijing, China; ^2^College of Pharmacy, Chengdu University of Traditional Chinese MedicineChengdu, China; ^3^Beijing Friendship Hospital, Capital Medical UniversityBeijing, China; ^4^Integrative Medicine Center, 302 Military HospitalBeijing, China

**Keywords:** *Polygonum multiflorum*, liver injury, bile acid, biomarker, LC-MS

## Abstract

Heshouwu (HSW), the dry roots of *Polygonum multiflorum*, a classical traditional Chinese medicine is used as a tonic for a wide range of conditions, particularly those associated with aging. However, it tends to be taken overdose or long term in these years, which has resulted in liver damage reported in many countries. In this study, the indicative roles of nine bile acids (BAs) were evaluated to offer potential biomarkers for HSW induced liver injury. Nine BAs including cholic acid (CA) and chenodeoxycholic acid (CDCA), taurocholic acid (TCA), glycocholic acid (GCA), glycochenodeoxycholic acid (GCDCA), deoxycholic acid (DCA), glycodeoxycholic acid (GDCA), ursodeoxycholic acid (UDCA), and hyodeoxycholic acid (HDCA) in rat bile and serum were detected by a developed LC-MS method after 42 days treatment. Partial least square-discriminate analysis (PLS-DA) was applied to evaluate the indicative roles of the nine BAs, and metabolism of the nine BAs was summarized. Significant change was observed for the concentrations of nine BAs in treatment groups compared with normal control; In the PLS-DA plots of nine BAs in bile, normal control and raw HSW groups were separately clustered and could be clearly distinguished, GDCA was selected as the distinguished components for raw HSW overdose treatment group. In the PLS-DA plots of nine BAs in serum, the normal control and raw HSW overdose treatment group were separately clustered and could be clearly distinguished, and HDCA was selected as the distinguished components for raw HSW overdose treatment group. The results indicated the perturbation of nine BAs was associated with HSW induced liver injury; GDCA in bile, as well as HDCA in serum could be selected as potential biomarkers for HSW induced liver injury; it also laid the foundation for the further search on the mechanisms of liver injury induced by HSW.

## Introduction

Heshouwu (HSW), the dry roots of *Polygonum multiflorum*, has been used in oriental counties for centuries, and is gaining popularity for treatment of many ailments, particularly those associated with aging (Park et al., 2011). Traditional Chinese herbal medicine ordinarily recommended the use of herbs in complex formulas, however both of them were considered as non-toxic medicine and were always taken as a single. In 2002, raw HSW and processed HSW were authorized as health food additive by the government of People's Republic of China. Then both of them were taken as tonic in varies forms in many countries (Yu et al., 2011; Dong et al., 2014).

However, hepatic adverse effect was constantly reported since 1990s in China, Canada, Korea, Japan, Britain, Italy, Australia, and other countries (Park et al., 2001; Lei et al., 2015), and supervisions of usage of HSW were conducted by drug regulatory agencies of Canada, British, Australia, and China, due to continually adverse effect reports. Therefore, detection of liver injury induced by HSW remains an issue to be tackled.

Currently, assessment for HSW induced liver injury relies primarily on histopathology and clinical chemistry based on a panel of serum biomarkers, including aspartate aminotransferase (AST), alanine aminotransferase (ALT), alkaline phosphatase(ALP), γ-glutamyltransferase (GGT) and total bilirubin (TBil) (Park et al., 2001; Jung et al., 2011; Dong et al., 2014). However, histopathological diagnosis only offers a destination indicator and may lack sufficient sensitivity. Changes with the common serum biomarkers may be contributed by factors other than HSW induced liver injury (Ennulat et al., 2010). These limitations have prompted the discovery of new biomarkers for HSW induced liver injury diagnosis, which will complement the current methodology. Therefore, detection of bile acids (BAs) might be an applicable approach in addressing detection of liver injury induced by HSW.

BAs are generated in the liver and accumulate in blood during liver injury, and have been proposed as biomarkers for liver injury and dysfunction (Reichel and Sauerbruch, 1995; Neghab and Stacey, 2000; Hofmann and Hagey, 2008). Furthermore, there were reports that identified perturbation of BA homeostasis as a common and early event of drug induced liver injury by diverse hepatotoxins (Yamazaki et al., 2013). Numerous BA species have been detected in mammals, and the primary BAs, cholic acid (CA) and chenodeoxycholic acid (CDCA), and their amino acid conjugate species and deoxycholic acids were most abundant in mice and man (de Aguiar Vallim et al., 2013).

In this study, nine BAs that were abundant in mice and man were simultaneously determined and evaluated for HSW-induced liver injury. Based on our previous study on the toxic dose of HSW, dosage and duration of administration time were determined (Li et al., 2013; Tu et al., 2015). LC-MS method was applied to quantified nine metabolites in rat bile and serum samples collected after the last administration, including CA, CDCA, taurocholic acid (TCA), glycocholic acid (GCA), glycochenodeoxycholic acid (GCDCA), deoxycholic acid (DCA), glycodeoxycholic acid (GDCA), ursodeoxycholic acid (UDCA), and hyodeoxycholic acid (HDCA). Chlorpromazine was selected as positive control as intrahepatic cholestasis is manifested in liver injury of reported studies (Regal et al., 1987). The indicative roles of the nine BAs were evaluated by partial least square-discriminate analysis (PLS-DA), and metabolism of the nine BAs was summarized, this study offered potential biomarkers for liver injury induced by HSW, which might be conductive to HSW's clinical application, and provide a basis for further mechanism study of liver injury induced by HSW.

## Materials and methods

### Chemicals, reagents, and materials

Raw HSW (10050904) and processed HSW (10122402) were purchased from the Lvye pharmaceutical co., ltd. (Beijing, China), and the materials were identified by Professor Xiao-he Xiao (PLA Institute of Chinese Material Medical, 302 Hospital of People's Liberation Army, Beijing, 100039, PR China). According to Chinese pharmacopeia (China Pharmacopoeia Committee, 2010), the quality of raw HSW and processed HSW were controlled. Chlorpromazine was purchased from Sigma (St. Quentin Fallavier, France).

Acetonitrile, methanol, formic acid were from Fisher Chemicals (Pittsburg, PA, USA). Ammonium acetate of HPLC grade was from Fluka (Buchs, Switzerland). Water was purified using a Milli-Q water purification system (Millipore, Bedford, MA, USA). Standards references of cholic acid (CA), chenodeoxycholic acid (CDCA), deoxycholic acid (DCA), glycochenodeoxycholic acid (GCDCA), hyodeoxycholic acid (HDCA), ursodeoxycholic acid (UDCA), taurocholic acid (TCA), glycocholic acid (GCA), glycodeoxycholic acid (GDCA), and bendrofluazide (IS) were purchased from the Control of Pharmaceutical and Biological Products (Beijing, China). Their purities were above 98%.

Preparation of herb extract: the dried and powdered raw HSW (1 kg) and processed HSW (1 kg) were respectively extracted five times by macerating with 6 L of 75% ethanol for 48 h each time. All the solvents were collected and then removed under reduced pressure, and HPLC was applied to multicomponent quantification of the extracts, which was depicted in Supplementary Material. Furthermore, a LC-MS method has been applied to analysis components of extracts of raw and processing HSW, giving more information about difference between raw and processing HSW, the methods and results (Figure S1 and Table S1) were depicted in Supplementary Material. Then, the extracts of raw HSW and processed HSW were redissolved with water to produce equivalent to 8 g of crude drug/mL, respectively.

### Animals, administration, and sample collection

Male Sprague-Dawley (SD) rats (160 ± 20 g) were obtained from the Academy of Military Medical Science (Beijing, China). All the animals were kept under the same laboratory conditions of temperature from 20 to 22°C and were given access to standard laboratory chow and tap water. The procedures involving animals and their care conform to the Guiding Principles for the Care and Use of Laboratory Animals of China. Prior to treatment, the rats were kept in the breeding room for 3 days and were randomly divided into four groups (*n* = 6 for each group). Group I was orally administrated with 5.0 mL of water everyday as the vehicle. Group II, III, and IV were orally administrated with 50.0 g/kg/day of processed HSW extract, 50.0 g/kg/day of raw HSW extract and 0.1 g/kg/day of chlorpromazine respectively for 42 days. Retro-orbital blood samples were collected into tubes every week for serum biochemical indicators detection. After last administration, 1.5 mL of blood sample was collected at time point of 1, 2, 3, and 4 h. The blood samples were immediately centrifuged at 3000 rpm (4°C) for 15 min to separate the serum. The serum collected at different time point from the same rat was mixed. Bile was collected after finishing the blood collection. The rat was anesthetized using midazolam (5 mg/kg) and the common bile duct was cannulated with a 30-gauge needle attached to PE-10 tubing. Bile was collected from the cannula for 4 h at 15-min intervals. The bile samples of one rat were mixed. The mixed samples were sub-aliquoted into the sample containers to be stored at 80°C until analysis. After bile sample collection, rats were sacrificed, and the change of liver histopathology was assessed.

### Analysis of liver injury biomarkers

#### LC-MS conditions

A Shimadzu (Japan) LC–MS 20AD system was carried out in the assay. The chromatographic separation was achieved on a Ulimate C_18_ column (150 mm × 4.6 mm, 5 μm). Methanol (containing 0.1% fomic acid, solvent A)—water (containing 1 mmol ammonium acetate and 0.1% fomic acid, solvent B) system was used as the mobile phase. The gradient elution condition was optimized as follows: linear gradient from 70 to 80% A (0–3 min), 80 to 90% A (3–8 min), 90 to 95% A (8–8.5 min), 95 to 100% A (8.5–14.4 min) then back to 70% A in 0.1 min, and maintain at 70% A for 5.5 min. The flow rate of the mobile phase was set at 0.7 mL/min, and 25% of the eluent was splitted into the inlet of the mass spectrometer. The column and autosampler temperature were maintained at 40°C and 4°C, respectively. The analytes and IS were equipped with electrospray ionization (ESI) interface in negative ion mode. The quantitative analysis was conducted by MRM at *m/z* 407.2 → 407.2 for CA, *m/z* 391.2 → 391.2 for CDCA, *m/z* 391.2 → 391.2 for DCA, *m/z* 464.2 → 74.0 for GCA, *m/z* 448.2 → 74.0 for GCDCA, *m/z* 448.2 → 74.0 for GDCA, *m/z* 391.3 → 391.3 for HDCA, *m/z* 514.2 → 79.8 for TCA, *m/z* 391.2 → 391.2 for UDCA, and *m/z* 419.95 → 419.95 for IS. The optimum source conditions were set as follows: nebulizing gas of 1.2 L/min; heat block temperature of 200°C; CDL temperature of 350°C; detector voltage of 1.75 kV; DP of 80 V and EP of 10 V.

#### Preparation of standard solutions and calibration curves

Ten mg/ml stock solutions of BAs and ISs were separately prepared in MeOH: water (1: 1, v/v). Bile and serum were collected from the 6 untreated mice, and bile was 100-fold diluted using deionized water. The diluted bile and serum were incubated with 100 mg/ml activated charcoal for 1 h to strip these matrices of endogenous BAs. Mixtures were centrifuged at 15,000 rpm for 10 min, and the supernatants were filtered. The filtrates were used to construct the calibration curves. Fixed volumes of these stripped matrices were spiked with 20 μL of the appropriate standard solution containing IS to construct a calibration curve with the range of 0.05–20 μg/mL, 0.01–20 μg/mL, 0.01–10 μg/mL, 0.01–100 μg/mL, 0.05–100 μg/mL, 0.01–20 μg/mL, 0.01–10 μg/mL, 0.01–20 μg/mL, 0.01–10μg/mL for CA, CDCA, DCA, GCA, GCDCA, GDCA, HDCA, TCA, UDCA, respectively. The concentration of the IS was 2 μg/mL.

#### Sample preparation

The blood and bile samples used in this study were collected after the last administration. An aliquot of 300 μL mixed serum sample spiked with 30 μL IS working solution, was added with 2700 μL methanol-acetonitrile (1: 1, v/v), vortexed for 1 min, and centrifuged at 9000 rpm for 10 min. The supernatant was aspirated, evaporated under vacuum, and reconstituted in 300 μL of MeOH. Bile samples were diluted 100-fold with deionized water, 100 μL of diluted bile samples were spiked with 10 μL IS working solution, and then added with 900 μL methanol-acetonitrile (1: 1, v/v), after vortexed mixing for 1 min, ultrasonic extraction for 5 min, and centrifugation for 10 min at 9000 rpm, The supernatant was aspirated, evaporated under vacuum, and reconstituted in 100 μL of MeOH.

#### Method validation

The limit of quantification (LOQ) was defined as the lowest concentrations with a signal-to-noise (S/N) ratio of 10:1. The method was validated using 5 QC points for each calibration curve. Five replicates of each QC point were analyzed repeatedly at 3 continuous days to determine the intra- and inter-day accuracy and precision. The concentrations of the QC points were 0.01, 0.1, 1, 10, 100 μg/mL for all BAs. Inter-day accuracy and precision were calculated from the % bias [% (Measured—Theoretical)/Measured concentrations] and relative standard deviation [% RSD = % Standard Deviation/Mean], respectively, for the 5 replicates of each QC point.

#### Data processing and statistical analysis

All values were indicated as mean ± standard deviation (mean ± SD). Differences between groups were tested by One-Way analysis of variance (ANOVA) using SPSS 20.0. To evaluate the indicative roles of the nine BAs, the concentration list data obtained by the MRM was used for multivariate analysis (PLS-DA, partial least square-discriminate analysis) using the SIMCA-P (version 11.0), after principal component analysis (PCA). The influence of raw HSW, processed HSW and chlorpromazine on the metabolism of BA was summarized, giving reference for further mechanism study of liver injury of HSW.

## Results

### Method validation

**Figure 1:**
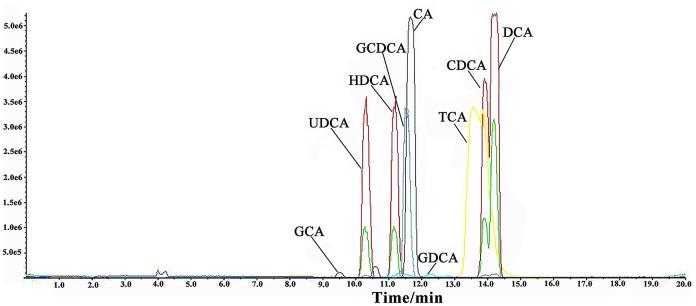
**LC/MS/MS chromatograms of nine bile acids**.

Nine BAs were simultaneously detected in rat bile and serum by LC-MS with MRM mode. LC/MS/MS chromatograms of the nine BAs were shown in Figure 1. The assay was validated according to the U.S. FDA guidance on bioanalytical method validation. To ensure the method reliability and reproducibility for BAs analysis, 5 QC concentrations distributed throughout the calibration range for each analyte in each matrix were determined to evaluate intra-day and inter-day accuracy and precision. The inter-day accuracy and precision for all analytes in bile were less than 10% (Table S2 depicted in Supplementary Material). The inter-day accuracy and precision for all analytes in serum was less than 10% at all concentration levels (Table S3 depicted in Supplementary Material). Good linearity was achieved over the calibration range (*r*^2^> 0.99), and calibration curves covered wide dynamic ranges. The LOQ for the nine BAs ranged between 10 and 50 ng/ml. Furthermore, the stability of stock solutions under storage conditions was tested. BAs were stable for at least 1 month in the −80°C freezer, and 10 days in the −20°C freezer respectively, stabilities of the analytes were proved acceptable (Tables S4, S5 depicted in Supplementary Material).

### Quantitative analysis of bile acids

The amounts of the nine BAs in bile and serum (except GDCA and TCA, which were not detected) were listed in Table 1. The analytes were quantitatively analyzed with the method described in the Experimental section. The concentrations of BAs in treatment groups were compared with normal control group by one-way analysis of variance (ANOVA) using SPSS 20.0. Despite big variations between individuals, significant difference was observed for BAs at specific treatment groups, as labeled in Table 1.

**Table 1:** **Bile and serum concentrations of bile acids in normal control, processed HSW, raw HSW, and chlorpromazine treated groups**.

**Compounds**	**Normal control**	**Processed HSW**	**Raw HSW**	**Chlorpromazine**
**BILE ACID (ng/ml, mean ± SD)**				
CA	125050.00±36668.08	167283.33±49153.64^*^	99050.00±41540.19^†^	68663.33±40810.28^††^
CDCA	884.17±256.17	1219.50±596.60^*^	475.42±242.32^††^	322.83±89.92^††^
DCA	380.17±66.76	211.57±90.00^†^	19.55±9.77^††^	39.89±22.67^††^
GCA	57883333.33±5299001.48	77400000.00±5091954.44^**^	55666666.67±8155529.82	21398333.33±8946105.53^††^
GCDCA	523166.67±64534.81	823833.33±147397.22^*^	456750.00±90797.54	125616.67±40574.58^††^
GDCA	202166.67±40341.57	213983.33±68986.23	43821.67±20048.25^††^	24390.00±13348.07^††^
HDCA	1260.33±320.75	611.50±169.02^†^	165.62±83.37^††^	327.67±103.45^††^
TCA	1268333.33±39168.44	1376666.67±26583.20^*^	1535000.00±58544.85^**^	1593333.33±71530.88^**^
UDCA	221.03±106.61	202.92±90.09^†^	98.32±25.83^††^	49.77±15.96^††^
**SERUM (ng/ml, mean ± SD)**				
CA	2210.40±1184.29	3227.50±1077.58	3023.50±1407.08^*^	3922.50±1112.73^*^
CDCA	1155.85±721.26	3017.00±1596.28	688.00±198.72	1032.30±394.29
DCA	224.98±98.56	173.50±56.52^†^	37.92±12.81^††^	81.89±27.24^††^
GCA	5788333.33±264950.08	7740000.00±254597.72	5566666.67±407776.49	2139833.33±447305.28^††^
GCDCA	628.00±83.85	102.50±50.20^††^	288.80±104.17^††^	358.65±82.72^†^
HDCA	825.25±333.65	636.10±184.25	20.45±7.69^††^	435.00±177.86
UDCA	49.81±15.65	96.44±36.07^*^	63.73±27.12^*^	83.11±40.51^*^

** P ≤ 0.01;

* *P ≤ 0.05, significantly increased*.

†† P ≤ 0.01;

† *P ≤ 0.05, significantly decreased*.

### Liver injury biomarker analysis

This study was the further study of screening for sensitive indicators of liver injury induced by HSW. The result of normal serum biochemical indicators and liver histopathology has been presented in our previous study (Tu et al., 2015). In that study, we have got the conclusion that processing could reduce the toxicity of HSW, and DBIL, TBIL were more sensitive than ALT, AST, and ALP.

**Figure 2:**
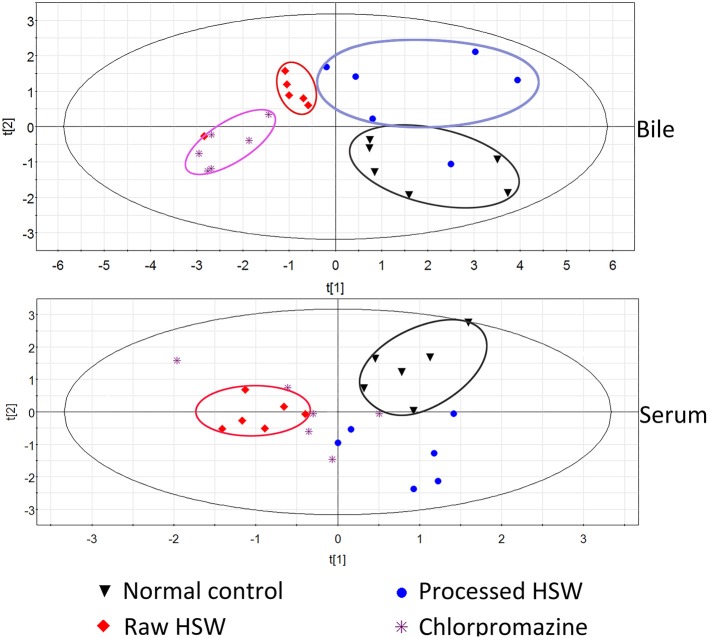
**PLS-DA clusters based on the quantitative analysis of BAs in rat bile and serum (***n*** = 6) from different treatment groups in negative ESI mode**.

To further investigate the indicative roles of the nine BAs, the concentration list data obtained was used for PLS-DA using the SIMCA-P (version 11.0) after PCA. The scatter plots were shown in Figure 2 and Figure S2. It was indicated that normal control group and raw HSW overdose treatment group were separately clustered and could be clearly distinguished with quantitative analysis of BAs in rat bile and serum, however, there was cross between normal control group and processed HSW overdose treatment group, between raw HSW overdose treatment group and chlorpromazine treatment group in the clusters. The result of clusters were consistent with the result of histopathology shown in our previous study, the liver section of normal control rats showed normal hepatocyte structure, and the histopathology of processed HSW group rat livers was similar to normal control group, no obvious hepatocyte necrosis was found. Mild to moderate hepatocyte necrosis and distinct hepatic sinusoid hyperemia occurred in rats receiving raw HSW. The hepatocyte necrosis and hepatic sinusoid hyperemia were much severe in rat receiving chlorpromazine (Tu et al., 2015).

**Figure 3:**
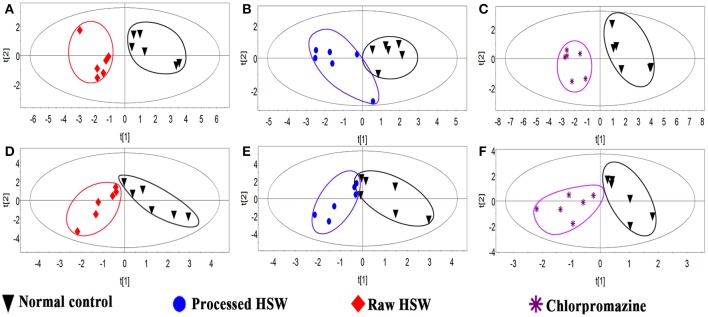
**Discrimination of normal control rats from raw HSW, processed HSW and chlorpromazine treatment rats according to PLS-DA**. **(A–C)** Discriminations based on the quantitative analysis of BAs in rat bile. **(D–F)** Discriminations based on the quantitative analysis of BAs in rat serum.

**Figure 4:**
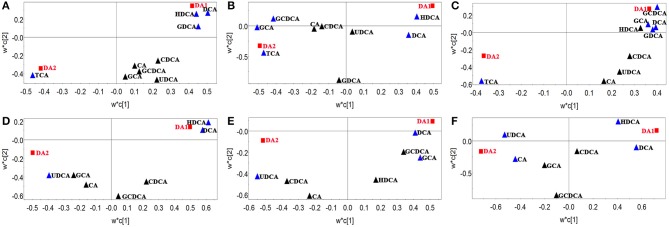
**Loading plots of PLS-DA between normal control group and raw HSW overdose treatment group, processed HSW overdose treatment group, chlorpromazine treatment group, respectively**. Loading plots of PLS-DA between normal control group and raw HSW overdose treatment group based on bile acids in rat bile **(A)** and rat serum **(D)**. Loading plots of PLS-DA between normal control group and processed HSW overdose treatment group based on bile acids in rat bile **(B)** and rat serum **(E)**. Loading plots of PLS-DA between normal control group and chlorpromazine treatment group based on bile acids in rat bile **(C)** and rat serum **(F)**. Blue triangles were BAs with VIP > 1 in the model.

In order to find characteristic biomarkers for liver injury induced by HSW, PLS-DA was applied to investigate deep differences between normal control group and raw HSW overdose treatment group, processed HSW overdose treatment group, chlorpromazine treatment group, respectively. The variable influence on the projection (VIP) parameter was applied to select variables that have significant contribution in clusters between two groups, the PLS-DA plots were shown in Figure 3, the loading plots were shown in Figure 4 and variables with VIP > 1 were marked with blue triangles, and the results of model validation plots were shown in Figure S3 depicted in Supplementary Material. The PLS-DA parameters including R^2^X, R^2^Y, Q^2^Y, R^2^-intercept and Q^2^-intercept were listed in Table S6. The PLS-DA clusters between two groups showed that normal control group and raw HSW treatment group could be distinguished with BAs in bile and also in serum. Normal control group and chlorpromazine treatment group could be distinguished with BAs in bile and also in serum. There was cross between normal control group and processed HSW treatment with BAs in bile and also in serum. The variables with VIP > 1 in clusters between normal control group and raw HSW treatment group were selected as potential biomarkers, as there was no obvious liver injury in process HSW treatment group, the variables with VIP > 1 in clusters between normal control group and process HSW treatment group were excluded, as a result GDCA in bile, as well as HDCA in serum were selected as potential biomarkers for HSW induced liver injury.

## Discussion

HSW is a traditional Chinese medicine, which has been used for a long time, and was traditional regarded as non-toxic. As far as we know, the first study focused on the toxicity of it was reported in 1996 (But et al., 1996), and at present the detection of it was mainly relied on the standard rules of common liver injury detection, however, this approach has reported to have limitation in sensitivity (Yamazaki et al., 2013). So it is urgently needed to find new biomarkers for HSW induced liver injury diagnosis to complement the current methodology. Synthesis, metabolism, clearance and intestinal absorption of BAs has been reported to enrolled in hepatic diseases (Bobeldijk et al., 2008; Alnouti, 2009), and total serum bile acid has been suggested to be sensitive than some traditional assays for liver disease (Bouchier and Pennington, 1978). Our previous study has showed that DBIL and TBIL were more sensitive than ALT, AST, and ALP to liver injury induced by HSW (Tu et al., 2015), and aqueous extracts of HSW with high dose has reported to decrease the content of TBIL in serum (Wang et al., 2012). Therefore, detection of BAs seems to be an advisable method to detect toxicity of HSW. Some previous research has shown that perturbation of bile acids present in multiple models pertaining to HSW (Bechmann et al., 2013; Péan et al., 2013; Luo et al., 2014). However, there was no report about analysis on individual bile acids in bile or serum in rats with liver injury induced by HSW.

In this assay, targeted analysis was developed for nine BAs that were abundant in mammals, to search for potential biomarkers for liver injury induced by HSW. GDCA in bile, as well as HDCA in serum were significant decrease and were selected as potential biomarkers for HSW induced liver injury. GDCA has been selected as biomarker in acetaminophen-induced acute liver failure in clinical study (Woolbright et al., 2014), Dietary HDCA exerted hypolipidemic effects by reducing farnesoid X receptor antagonist bile acids in mouse enterohepatic tissues (Watanabe and Fujita, 2014). Bile acids was synthesized in liver, and transported from hepatocytes into the biliary tracts by canalicular transporters, this process was sensitive to perturbation (Jaeschke et al., 2002; Yamazaki et al., 2013), which further confirmed that bile acids were highly sensitive markers for liver injury. Although the mechanism behind why specific bile acid was elevated or decreased in liver injury rats was not solved in this paper, and the potential biomarkers needed further validation on clinical stage, the value of examining individual BAs was emphasized, which with a clinical study might offer a new potential for early warning, diagnosis and prognosis of liver injury induced by HSW.

**Figure 5:**
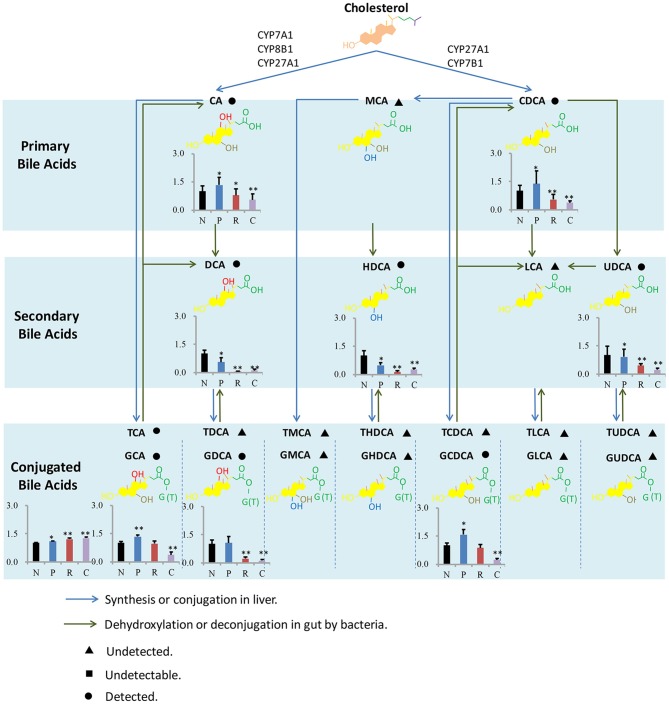
**Bile acids metabolism in liver and intestine**. The bar graphs show the differences of concentrations of BAs between four groups, and the concentrations of BAs in normal control group were set as 1 as the control. N, P, R, and C represent the normal control, processed HSW, raw HSW and chlorpromazine group separately. ^**^P ≤ 0.01; ^*^P ≤ 0.05, significantly change was observed when compared with normal control.

As far as we known, present study about liver injury induced by HSW was mostly focused on clinical case report and hepatotoxic components in HSW (Lin et al., 2015), except for a little *in vitro* assays focused on L02 cell and P450 enzyme (Unger and Frank, 2004), there was little mechanism study of liver injury induced by HSW. In order to provide some information for further mechanism study of liver injury induced by HSW, metabolism of BA was summarized, and shown in Figure 5. BAs were derived from the catabolism of cholesterol, and BAs pool could be divided into primary and secondary BAs based on biological source. In the progress of primary BAs biosynthesis, P450 enzymes including CYP7A1, CYP8B1, CYP27A1, and CYP7B1 were involved in de Aguiar Vallim et al. (2013), and there has been literatures reporting that HSW and anthraquinones in it would inhibit P450s involved in metabolism of pharmaceuticals (Sridhar et al., 2012; Wang et al., 2015). However, there was little study focusing on the influence of HSW to P450s involved in primary BAs biosynthesis in pathogenesis of liver injury. As present study has showed significant change of primary bile acids, it would be meaningful to investigate the activity of related P450s in further study. In addition, we have detected significant change of secondary bile acids followed the ingestion of HSW. It is known the biosynthesis of secondary bile acids is tied to gut microorganisms (Liu et al., 2015). Nevertheless, anthraquinones, one of the major types of components in HSW, have been reported to own strong antibacterial effect (Njeru et al., 2015). And anthraquinones could induce diarrhea and further alter the gut microorganism composition (David et al., 2015; Goulet, 2015). Hence, in the further study, it would be interesting to investigate the role of gut microorganisms played in liver injury induced by HSW.

We acknowledge several limitations with this study. First, we have only analyzed a limited number of BAs. It will be necessary to validate these findings with a more extensive panel. Second, we need to further extend the coverage of metabolites by the untargeted metabolomic platform to assess if more sensitive DILI biomarkers can be discovered. Third, the dose of HSW used this study referred to HSW used in the assay of subacute toxicity (Wu et al., 2012), equivalent to 100 times of the upper dose of human stipulated in Chinese Pharmacopoeia (Wang et al., 2012), and histopathology of these rats has been studied to confirm that liver injury was appeared in raw HSW and chlorpromazine group, however the condition was similar but not equal to clinical condition. For the idiosyncratic hepatotoxins, more doses and time points at a clinical stage need to be detected to establish the clinical relevance to humans.

### Conflict of interest statement

The authors declare that the research was conducted in the absence of any commercial or financial relationships that could be construed as a potential conflict of interest.
