# Supplementary material for: Screening for biomarkers of liver injury induced by Polygonum multiflorum: a targeted metabolomic study
Source: Front Pharmacol. 2015 Oct 2;6:217. doi: 10.3389/fphar.2015.00217 (PMC4591842; doi:10.3389/fphar.2015.00217)
Supplement: Supplementary file 1 [file DataSheet1.DOCX]

**Supplementary material**

**Screening for biomarkers of liver injury induced by *Polygonum multiflorum*: a targeted** **metabolomic study**

Qin Dong ^1, 2^, Na Li ^1^, Qi Li ^1^, Cong-en Zhang ^1, 2^, Wu-wen Feng ^1, 2^, Guang-quan Li ^1, 2^, Rui-yu Li ^1, 2^, Can Tu ^1^, Xue Han ^1, 2^, Zhao-fang Bai ^1^, Ya-ming Zhang ^1^, Ming Niu ^1^, Zhi-jie Ma ^3,^ *, Xiao-he Xiao ^4,^ *, Jia-bo Wang ^1,^ *

^1^ *China Military Institute of Chinese Medicine,* *302 Military Hospital, Beijing , China*

^2^ *College of Pharmacy, Chengdu University of Traditional Chinese Medicine, Chengdu , China*

^3^ *Beijing Friendship Hospital, Capital Medical University, Beijing, China*

*^4^ Integrative Medicine Center, 302 Military Hospital, Beijing, China*

Correspondence:

Jia-bo Wang and Xiao-he Xiao,

302 Military Hospital, No. 100 Xisihuan Beijing 100039, China

Fax: +86 66933322; +86 66933325

E-mail: pharm_sci@126.com; pharmacy302xxh@126.com

Zhi-jie Ma,

Beijing Friendship Hospital, No. 95 Yongan Beijing 100039, China

Fax: +86 63139287

E-mail: [13811647091@163.com](mailto:13811647091@163.com)^[[1]](#footnote-1)^

**Multicomponent quantification of the raw and processed *Polygonum multiflorum* extracts by HPLC**

Analyses were performed using an Agilent 1200 HPLC system (Agilent Technologies, Santa Clara, California, USA). Chromatography was carried out at 30ºC on a Agilent Eclipse Plus C_18_ column (250mm×4.6mm, with 5 μm particle size). The mobile phase consisted of methanol (A) and 0.01% (v/v) phosphoric acid water solution (B). The gradient elution was 20-30% A from 0-10 min, 30-40% A from 10 to 15 min, 40-50% A from 15 to 25 min, 50-60% A from 25 to 30 min, 60-70% A from 30 to 40 min, 70-80% A from 40 to 45 min, and 80-85% A from 45 to 50 min, at a flow rate of 1.0 mL⋅min^−1^. The signal was monitored at 254 nm.

Standards of 2,3,5,4-Tetrahydroxystilbene-2-O-b-D-glucoside, emodin, emodin-8-O-glycosidase, gallic acid, catechin and physcion were purchased from the National Institute for the Control of Pharmaceutical and Biological Products (Beijing, China) and the purity of all these compounds was higher than 98.0%.

HPLC-grade methanol was purchased from Fish Chemicals (Pittsburg, USA). Water was purified by a Milli-Q Plus water purification system (Millipore, USA). AR-grade phosphoric acid was obtained from Beijing Chemical Factory, Beijing.

The concentrations of 2,3,5,4-Tetrahydroxystilbene-2-O-b-D-glucoside, emodin, emodin-8-O-glycosidase, catechin and physcion in raw *Polygonum multiflorum* (Raw HSW) was 17.11%, 0.58%, 2.2%, 1.02%, and 0.55% separately, and the concentrations of these six components in processed *Polygonum multiflorum* (Processed HSW) was 10.11%, 0.43%, 1.2%, 0.92%, and 0.42% separately.

**LC-MS identification of components of the extracts of raw and processed *Polygonum multiflorum***

The extracts of raw and processed HSW preserved in our laboratory were accurately weighted and dissolved in 75% ehanol to construct a concentration of 5 mg/ml. Then, they were centrifuged at 13000 rpm for 15 min to separate the supernatant for LC-MS analysis.

Analysis was performed on a Agilent Acquity Ultra-performance LC system coupled with a iFunnel Q-TOF mass spectrometry equipped with a Dual AJS electrospray ionization (ESI) source (Agilent, Santa Clara, CA USA). The separation of all samples was performed on a Agilent Zorbax 300SB-C18 column (100 × 2.1 mm, 1.8 μm). The gradient elution employed acetonitrile as solvent A and water as solvent B. While maintaining a constant flow rate of 0.2 mL/min, the gradient program was as follows: 0-5 min, 5-32% A; 5-6 min, 32-55% A; 6-12 min, 55-85% A; 12-13 min, 85-90% A; 13-15 min, 90% A. The detection wavelength was 280 nm. The column temperature was kept at 30^◦^C.

The mass spectrometric data were collected with negative ionization. The gas temperature was set to 200^◦^C with a gas flow of 14 L/min, the sheath temperature of 350^◦^C and its gas flow of 11 L/min. The capillary voltage was 3.5 kV for negative ionization mode while the nebulizer was 35 psi. For full-scan MS analysis, the spectra were recorded in the range of *m/z* 100-3000. Leucine-encephalin at a concentration of 25 ng/L was used as the lock mass in negative mode ([M – H] ^−^ = 554.2615). Compounds were identified based on accurate mass and referring to the authentic standards or tentatively identified based on accurate mass and referring to related literatures. The mass data and compounds identified from the peaks are summarized in Fig. S1 and Table S1.


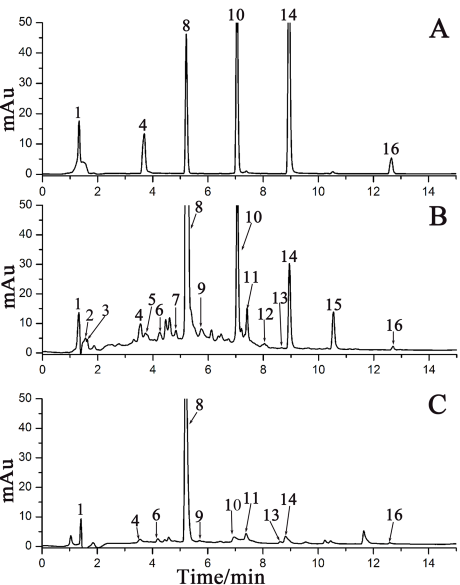


Fig. S1 UV chromatograms of mixed standards (A), raw HSW (B) and processed HSW at 280 nm. Peak numbers are consistent with those shown in Table S1

Table S1 Identified compounds in the extracts of raw HSW and processed HSW

| Peak | Time (min) | Raw HSW | |  | Processed HSW | | Molecular formula | Exact mass（Da） | Identification | Source |
| --- | --- | --- | --- | --- | --- | --- | --- | --- | --- | --- |
|  |  | Determined (m/z) | Error (ppm) |  | Determined (m/z) | Error (ppm) |  |  |  |  |
| 1 | 1.32 | 169.0135 | -4.1 |  | 169.0150 | 4.7 | C_7_H_6_O_5_ | 170.0215 | Gallic acid ^a)^ | R, P |
| 2 | 1.50 | 419.1697 | -3.3 |  | / | / | C_22_H_28_O_8_ | 420.1784 | *cis*-rhaponitin ^b)^ | R |
| 3 | 1.52 | 419.1692 | -4.5 |  | / | / | C_22_H_28_O_8_ | 420.1784 | Rhaponitin ^b)^ | R |
| 4 | 3.52 | 289.0711 | -2.1 |  | 289.0720 | 1.0 | C_15_H_14_O_6_ | 290.0790 | Catechin ^a)^ | R, P |
| 5 | 3.55 | 289.0701 | -5.5 |  | / | / | C_15_H_14_O_6_ | 290.0790 | L-Epicatechin ^b)^ | R |
| 6 | 4.25 | 729.1475 | 1.9 |  | 729.1481 | 2.7 | C_37_H_30_O_16_ | 730.1534 | Mono-0-galloylprocyanidin ^b)^ | R, P |
| 7 | 4.75 | 405.1180 | -2.7 |  | / | / | C_20_H_22_O_9_ | 406.1264 | cis-2,3,5,4'-Tetrahydroxystilbene-2-O-glc ^b)^ | R |
| 8 | 5.20 | 405.1192 | 0.2 |  | 405.1176 | -3.7 | C_20_H_22_O_9_ | 406.1264 | 2,3,5,4'-Tetrahydroxystilbene-2-O-glc ^a)^ | R, P |
| 9 | 5.63 | 557.1293 | -1.3 |  | 557.1302 | 0.4 | C_27_H_26_O_13_ | 558.1373 | Tetrahydroxystilbene-O-(galloyl)-hex ^b)^ | R, P |
| 10 | 7.20 | 431.0985 | 0.5 |  | 431.0973 | -2.3 | C_21_H_20_O_10_ | 432.1056 | Emodin-8-O-glc ^a)^ | R, P |
| 11 | 7.50 | 407.1333 | -3.4 |  | 407.1336 | -2.7 | C_20_H_24_O_9_ | 408.1420 | Torachrysone-8-O-glc ^b)^ | R, P |
| 12 | 8.00 | 559.1442 | -2.7 |  | / | / | C_27_H_28_O_13_ | 560.1530 | Torachrysone-O-glucogallin ^b)^ | R |
| 13 | 8.50 | 283.0603 | -3.2 |  | 283.0597 | -5.3 | C_16_H_12_O_5_ | 284.0685 | Anthraquinone ^b)^ | R, P |
| 14 | 9.13 | 269.0452 | -1.1 |  | 269.0443 | -4.5 | C_15_H_10_O_5_ | 270.0528 | Emodin ^a)^ | R, P |
| 15 | 10.50 | 277.1805 | -1.4 |  | / | / | C_17_H_26_O_3_ | 278.1882 | Heptadecanoic acid-ester ^b)^ | R |
| 16 | 12.70 | 283.0605 | -2.5 |  | 283.0615 | 1.1 | C_16_H_12_O_5_ | 284.0685 | Physcion ^a)^ | R, P |

R: raw HSW, P: processed HSW, “/”no useful information collected. a) Validated with standard sample. b) Tentatively identified according to literature.

**Method validation**

Table S2 Summary of the inter-day accuracy and precision for all analytes in rat bile

| Nominal Conc. | QC1 | |  | QC2 | |  | QC3 | |  | QC4 | |  | QC5 | |
| --- | --- | --- | --- | --- | --- | --- | --- | --- | --- | --- | --- | --- | --- | --- |
|  | 10 (ng/ml) | %R.S.D. |  | 100 （ng/ml） | %R.S.D. |  | 1 （μg/ml） | %R.S.D. |  | 10 (μg/ml) | %R.S.D. |  | 100 (μg/ml) | %R.S.D. |
| CA | 10.12 | 8.10 |  | 109.14 | 9.50 |  | 0.93 | 8.91 |  | 9.84 | 8.32 |  | 108.21 | 6.54 |
| CDCA | 9.34 | 9.91 |  | 108.25 | 9.87 |  | 1.04 | 9.10 |  | 9.21 | 7.10 |  | 105.34 | 5.69 |
| DCA | 9.13 | 9.65 |  | 95.89 | 9.10 |  | 0.98 | 7.24 |  | 10.67 | 7.45 |  | 103.24 | 7.23 |
| GCA | 10.58 | 9.21 |  | 97.21 | 7.51 |  | 0.91 | 8.02 |  | 10.21 | 9.21 |  | 92.11 | 8.21 |
| GCDCA | 9.87 | 8.32 |  | 105.36 | 9.00 |  | 1.09 | 9.01 |  | 10.00 | 6.55 |  | 90.98 | 9.00 |
| GDCA | 10.67 | 7.89 |  | 105.62 | 8.79 |  | 1.02 | 9.29 |  | 9.36 | 7.98 |  | 106.22 | 9.34 |
| HDCA | 9.56 | 9.00 |  | 92.31 | 9.00 |  | 0.91 | 7.21 |  | 10.24 | 6.97 |  | 95.45 | 8.76 |
| TCA | 10.21 | 8.34 |  | 91.51 | 8.12 |  | 1.05 | 9.35 |  | 9.52 | 8.21 |  | 105.67 | 8.21 |
| UDCA | 10.35 | 9.11 |  | 92.37 | 8.56 |  | 1.07 | 8.23 |  | 9.31 | 6.89 |  | 93.46 | 9.11 |

Table S3 Summary of the inter-day accuracy and precision for all analytes in rat serum

| Nominal Conc. | QC1 | |  | QC2 | |  | QC3 | |  | QC4 | |  | QC5 | |
| --- | --- | --- | --- | --- | --- | --- | --- | --- | --- | --- | --- | --- | --- | --- |
|  | 10 (ng/ml) | %R.S.D. |  | 100 (ng/ml) | %R.S.D. |  | 1 (μg/ml) | %R.S.D. |  | 10 （μg/ml） | %R.S.D. |  | 100 （μg/ml） | %R.S.D. |
| CA | 10.00 | 7.89 |  | 105.21 | 8.23 |  | 0.99 | 9.25 |  | 10.00 | 9.00 |  | 105.67 | 5.43 |
| CDCA | 9.21 | 9.88 |  | 102.34 | 9.24 |  | 0.91 | 8.77 |  | 9.87 | 8.21 |  | 104.00 | 7.32 |
| DCA | 9.03 | 9.24 |  | 101.00 | 9.10 |  | 0.95 | 9.30 |  | 9.43 | 9.11 |  | 106.32 | 6.98 |
| GCA | 10.54 | 8.90 |  | 106.74 | 8.73 |  | 1.04 | 8.34 |  | 9.01 | 8.90 |  | 92.31 | 9.00 |
| GCDCA | 10.21 | 8.76 |  | 94.53 | 7.90 |  | 1.01 | 9.01 |  | 9.22 | 8.76 |  | 90.67 | 4.67 |
| GDCA | 9.56 | 9.01 |  | 93.21 | 9.34 |  | 1.08 | 9.88 |  | 10.21 | 6.60 |  | 97.32 | 2.56 |
| HDCA | 9.21 | 8.56 |  | 91.15 | 9.00 |  | 0.91 | 7.89 |  | 10.55 | 7.21 |  | 90.93 | 9.34 |
| TCA | 10.34 | 9.00 |  | 103.22 | 8.45 |  | 0.92 | 9.23 |  | 9.45 | 7.98 |  | 103.21 | 8.32 |
| UDCA | 9.13 | 9.67 |  | 104.56 | 9.65 |  | 0.95 | 6.90 |  | 9.89 | 8.56 |  | 97.36 | 4.76 |

Table S4 Stability of the bile acids in rat bile

| Analyte | Low concentration (ng/mL) | | | | | | |  | High concentration (μg/mL) | | | | | | |
| --- | --- | --- | --- | --- | --- | --- | --- | --- | --- | --- | --- | --- | --- | --- | --- |
|  | NC | 10 d MC | 10 d V | %R.S.D. | 30 d MC | 30 d V | %R.S.D. |  | NC | 10 d MC | 10 d V | %R.S.D. | 30 d MC | 30 d V | %R.S.D. |
| CA | 10.00 | 10.35 | 3.50 | 7.89 | 9.05 | -9.50 | 9.82 |  | 100.00 | 108.21 | 8.21 | 8.30 | 92.13 | -7.87 | 9.00 |
| CDCA | 10.00 | 9.22 | -7.80 | 8.90 | 9.33 | -6.70 | 8.90 |  | 100.00 | 105.44 | 5.44 | 4.15 | 95.24 | -4.76 | 8.19 |
| DCA | 10.00 | 10.78 | 7.80 | 9.21 | 9.80 | -2.00 | 8.24 |  | 100.00 | 97.22 | -2.78 | 6.12 | 91.22 | -8.78 | 7.54 |
| GCA | 10.00 | 10.32 | 3.20 | 7.80 | 10.12 | 1.20 | 8.00 |  | 100.00 | 101.33 | 1.33 | 7.01 | 92.34 | -7.66 | 7.31 |
| GCDCA | 10.00 | 9.23 | -7.70 | 9.00 | 9.33 | -6.70 | 9.32 |  | 100.00 | 94.34 | -5.66 | 5.40 | 94.56 | -5.44 | 6.09 |
| GDCA | 10.00 | 9.06 | -9.40 | 8.13 | 9.14 | -8.60 | 8.45 |  | 100.00 | 103.56 | 3.56 | 6.45 | 92.69 | -7.31 | 9.01 |
| HDCA | 10.00 | 10.79 | 7.90 | 9.11 | 10.05 | 0.50 | 9.16 |  | 100.00 | 98.01 | -1.99 | 8.00 | 98.00 | -2.00 | 8.44 |
| TCA | 10.00 | 9.12 | -8.80 | 6.75 | 9.23 | -7.70 | 8.65 |  | 100.00 | 103.00 | 3.00 | 9.11 | 101.00 | 1.00 | 6.55 |
| UDCA | 10.00 | 10.66 | 6.60 | 7.65 | 9.15 | -8.50 | 7.95 |  | 100.00 | 92.99 | -7.01 | 8.76 | 92.72 | -7.28 | 7.7 |

Table S5 Stability of the bile acids in rat serum

| Analyte | Low concentration (ng/mL) | | | | | | |  | High concentration (μg/mL) | | | | | | |
| --- | --- | --- | --- | --- | --- | --- | --- | --- | --- | --- | --- | --- | --- | --- | --- |
|  | NC | 10 d MC | 10 d V | %R.S.D. | 30 d MC | 30 d V | %R.S.D. |  | NC | 10 d MC | 10 d V | %R.S.D. | 30 d MC | 30 d V | %R.S.D. |
| CA | 10.00 | 10.81 | 8.10 | 7.55 | 9.71 | -2.90 | 9.00 |  | 100.00 | 109.21 | 9.21 | 5.40 | 90.98 | -9.02 | 8.31 |
| CDCA | 10.00 | 10.78 | 7.80 | 9.00 | 9.05 | -9.50 | 8.98 |  | 100.00 | 105.34 | 5.34 | 6.79 | 93.46 | -6.54 | 5.01 |
| DCA | 10.00 | 10.62 | 6.20 | 8.76 | 9.33 | -6.70 | 9.71 |  | 100.00 | 92.00 | -8.00 | 9.00 | 97.00 | -3.00 | 6.91 |
| GCA | 10.00 | 9.01 | -9.90 | 9.67 | 10.01 | 0.10 | 9.21 |  | 100.00 | 95.44 | -4.56 | 7.93 | 91.45 | -8.55 | 7.00 |
| GCDCA | 10.00 | 9.34 | -6.60 | 8.90 | 9.55 | -4.50 | 9.40 |  | 100.00 | 98.00 | -2.00 | 8.59 | 98.21 | -1.79 | 5.56 |
| GDCA | 10.00 | 10.76 | 7.60 | 9.11 | 9.26 | -7.40 | 8.06 |  | 100.00 | 101.24 | 1.24 | 9.00 | 90.79 | -9.21 | 9.00 |
| HDCA | 10.00 | 10.79 | 7.90 | 9.38 | 9.51 | -4.90 | 7.89 |  | 100.00 | 95.61 | -4.39 | 8.34 | 92.11 | -7.89 | 4.67 |
| TCA | 10.00 | 9.34 | -6.60 | 7.88 | 9.09 | -9.10 | 8.90 |  | 100.00 | 105.29 | 5.29 | 7.98 | 93.14 | -6.86 | 7.25 |
| UDCA | 10.00 | 10.76 | 7.60 | 8.06 | 9.69 | -3.10 | 9.33 |  | 100.00 | 94.21 | -5.79 | 9.51 | 92.00 | -8.00 | 6.77 |

NC, nominal concentration; MC, measured concentration; V, variation in %; RSD, relative standard deviation in %; 10 d, 10 days storage; 30 d, 30 days storage

**Liver injury biomarker analysis**
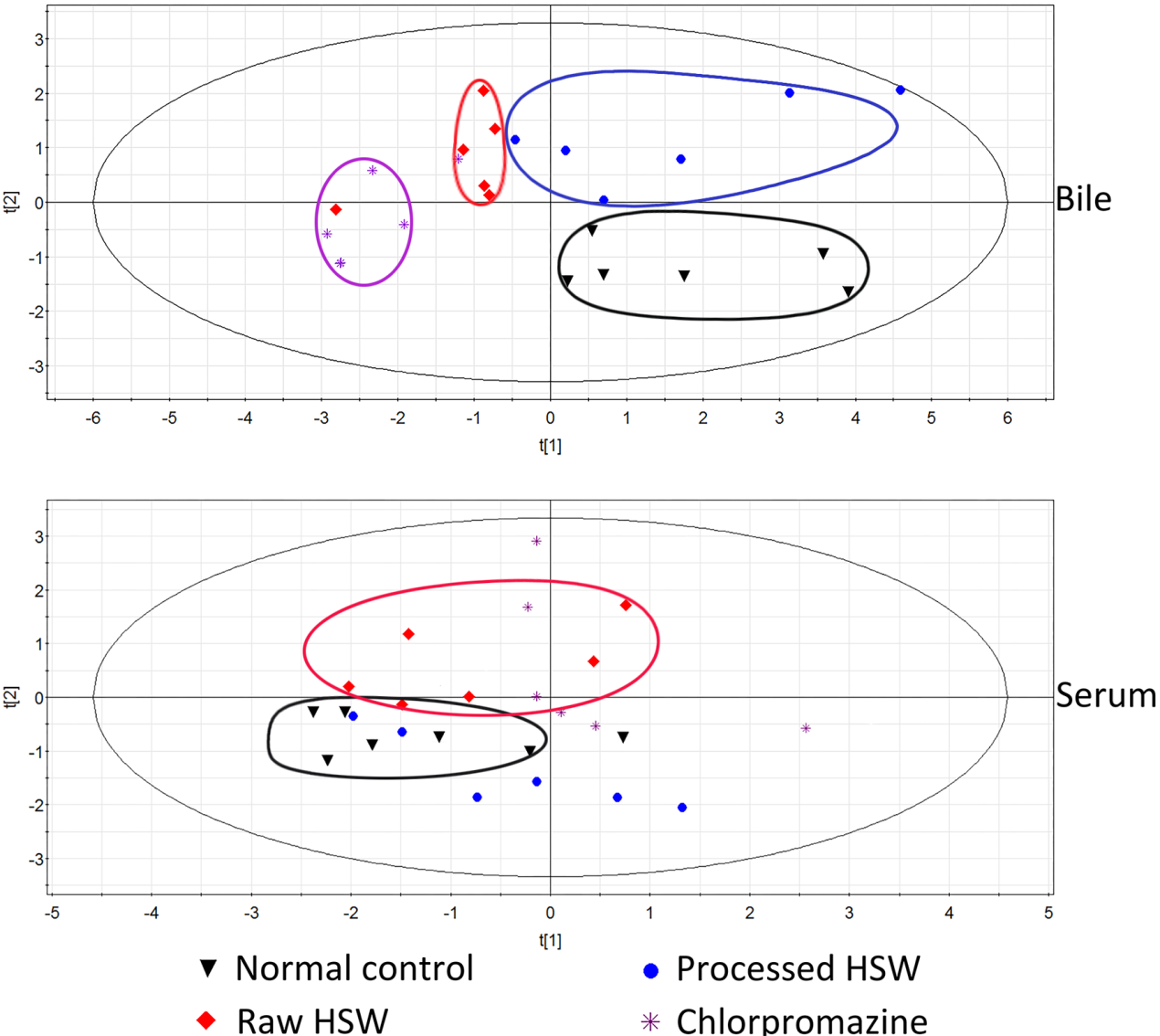


Fig. S2. PCA clusters based on the quantitative analysis of BAs in rat bile and serum (n=6) from different treatment groups in negative ESI mode.

The unsupervised principal component analysis (PCA) analysis reveals that the four groups were partly aggregated in bile sample. Normal control group and raw HSW overdose treatment group were separately clustered in serum sample. However this change is in a diversification manner. Partial least square-discriminate analysis (PLS-DA) was employed for classification or discrimination analyses in the manuscript because PLS-DA is a supervised analysis, and significant differences of biological variation could easily be detected.


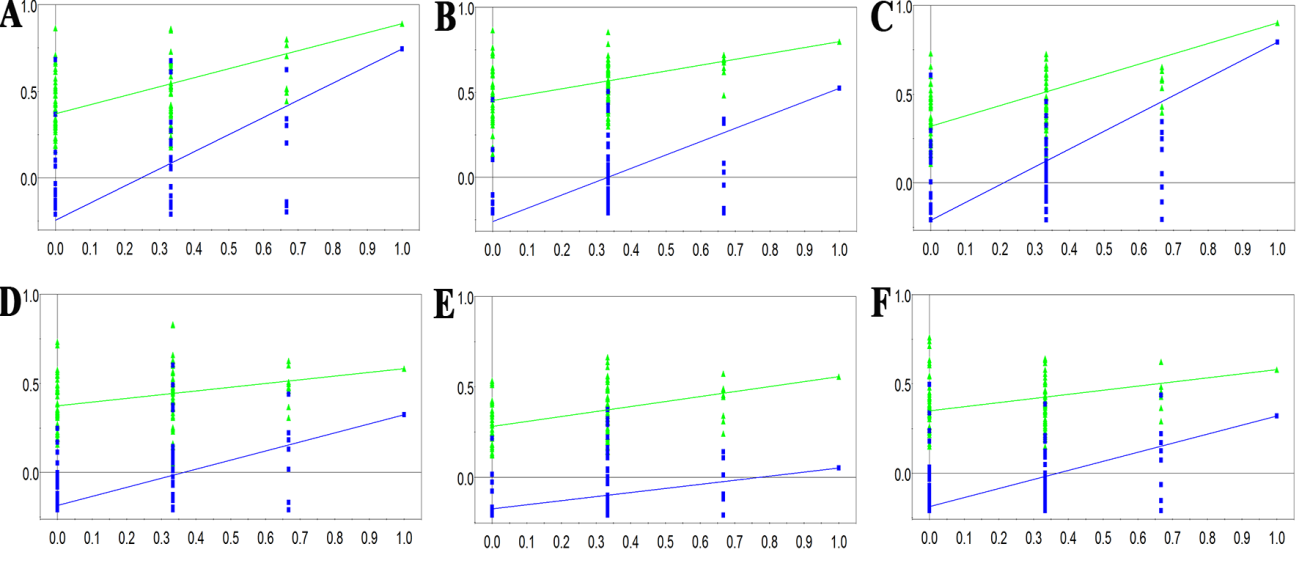


Fig. S3 Shown is 100-permutation test of PLS-DA between normal control group and raw HSW overdose treatment group, processed HSW overdose treatment group, chlorpromazine treatment group, respectively. The plot letter is consistent with the plot letter show in Figure 3.

Table S6 Parameters of PLS-DA models

| Model | Component | R^2^X | R^2^Y | Q^2^Y | R^2^-intercept | Q^2^-intercept |
| --- | --- | --- | --- | --- | --- | --- |
| A | 2 | 0.65 | 0.89 | 0.75 | 0.37 | -0.24 |
| B | 2 | 0.48 | 0.80 | 0.52 | 0.45 | -0.26 |
| C | 2 | 0.84 | 0.90 | 0.80 | 0.32 | -0.21 |
| D | 2 | 0.81 | 0.58 | 0.50 | 0.37 | -0.18 |
| E | 2 | 0.79 | 0.56 | 0.05 | 0.28 | -0.18 |
| F | 2 | 0.59 | 0.66 | 0.52 | 0.35 | -0.19 |

The letter of model is consistent with the letter in Figure 3, R^2^-intercept and Q^2^-intercept represent result of permutation testing.

1. [↑](#footnote-ref-1)
